# Comments on “Association Between F‐SIRI and Adverse Prognosis in Patients With Chronic Heart Failure”

**DOI:** 10.1002/clc.70181

**Published:** 2025-07-21

**Authors:** Çağrı Zorlu

**Affiliations:** ^1^ Department of Cardiology Tokat Gaziosmanpasa University Hospital Tokat Turkey

## Conflicts of Interest

The author declares no conflicts of interest.

Dear Editor,

1

We read with interest the article by Liu et al. published in Clinical Cardiology (2025; DOI: 10.1002/clc.70166), which explores the prognostic value of the fibrinogen and systemic inflammation response index (F‐SIRI) in patients with chronic heart failure (CHF) [1] The study provides valuable insights into the role of inflammation and coagulation in CHF risk stratification. However, we would like to raise several points for clarification and discussion to enhance the interpretation of the findings.

## 2 Methodological Concerns Regarding F‐SIRI Cut‐Off Values

The authors determined F‐SIRI cut‐off values (SIRI ≥ 1.22, fibrinogen ≥ 2.55 g/L) using a Receiver Operating Characteristic (ROC) curve with 100 random splits and threefold cross‐validation. While this approach is robust, the rationale for selecting these specific cut‐offs and their generalizability across diverse CHF populations is unclear. Could the authors provide further details on how these thresholds were validated externally or compared to existing literature? For instance, studies like Xia et al. used different SIRI cut‐offs for cardiovascular outcomes, which may affect the reproducibility of F‐SIRI [2]. Additionally, the single measurement of F‐SIRI at admission may not capture dynamic inflammatory changes, as acknowledged in the limitations. Have the authors considered serial measurements to assess the stability of F‐SIRI's prognostic value?

## 3 Inconsistency in Outcome Associations

The study reports a significant association between higher F‐SIRI levels and all‐cause mortality (adjusted HR 2.37, 95% CI 1.46–3.83, *p* < 0.001) but no significant association with major adverse cardiac and cerebral events (MACCEs) or cardiovascular death after multivariate adjustments. This discrepancy is puzzling, as inflammation and coagulation are established contributors to cardiovascular events in CHF [3, 4]. For example, the CANTOS trial demonstrated that targeting inflammation reduces cardiovascular events in patients with elevated inflammatory markers [5]. Could the authors elaborate on potential reasons why F‐SIRI predicts all‐cause mortality but not cardiovascular‐specific outcomes? Specifically, were non‐cardiovascular causes of death (e.g., infections and malignancies) analyzed separately to explain this finding?

## 4 Potential Confounding Factors

The multivariate Cox models adjusted for numerous confounders, including demographics, comorbidities, and medications. However, the study does not account for the severity of heart failure, such as New York Heart Association (NYHA) functional class or specific etiologies (e.g., ischemic vs. non‐ischemic CHF). These factors significantly influence prognosis and inflammatory markers [6]. Could the authors clarify whether NYHA class or heart failure etiology was considered in the analysis? Additionally, the lack of significant differences in left ventricular ejection fraction (LVEF) across F‐SIRI groups (*p* = 0.3596) is surprising, given the known association between inflammation and reduced LVEF in heart failure with reduced ejection fraction (HFrEF) [7]. Could the authors discuss whether the inclusion of both HFrEF and heart failure with preserved ejection fraction (HFpEF) patients might have diluted this association?

## 5 Statistical and Reporting Issues

The article reports identical event rates for all‐cause death and cardiovascular death across F‐SIRI groups (e.g., 24 [6.94%] in F‐SIRI = 0, 82 [11.19%] in F‐SIRI = 1, and 101 [19.80%] in F‐SIRI = 2 for both outcomes). This is likely a reporting error, as all‐cause death should encompass cardiovascular and non‐cardiovascular deaths. Could the authors confirm whether this is a typographical error or provide clarification on the data? Furthermore, the restricted cubic spline analysis suggests a non‐linear relationship between SIRI and outcomes, yet the discussion does not explore the clinical implications of this finding. Could the authors elaborate on how this non‐linearity might affect the practical application of F‐SIRI in risk stratification?

## 6 Comparison With Existing Inflammatory Markers

The authors highlight F‐SIRI's incremental predictive value over traditional risk factors but do not compare its performance to established inflammatory markers like C‐reactive protein (CRP) or interleukin‐6 (IL‐6), which are widely studied in CHF [8]. Given that F‐SIRI is a composite marker, a head‐to‐head comparison with CRP or other indices (e.g., neutrophil‐to‐lymphocyte ratio) would strengthen the claim of its superior prognostic utility. Have the authors considered such comparisons, and if not, could they discuss the feasibility of including these markers in future studies?

In conclusion, Liu et al.'s study is a commendable step toward integrating inflammation and coagulation markers for CHF prognostication. However, addressing the above points could enhance the robustness and clinical applicability of F‐SIRI. We encourage the authors to clarify these aspects and consider prospective studies to validate their findings. We appreciate the opportunity to engage in this scientific discussion and thank the authors for their contribution to the field.

## Data Availability

The author have nothing to report.
